# Premature CD4^+^ T Cells Senescence Induced by Chronic Infection in Patients with Acute Coronary Syndrome

**DOI:** 10.14336/AD.2020.0203

**Published:** 2020-12-01

**Authors:** Ming Cao, Lei Ruan, Yi Huang, Jinli Wang, Jinhua Yan, Yu Sang, Shanshan Li, Guan Wang, Xiaofen Wu

**Affiliations:** Department of Gerontology, Tongji Hospital, Tongji Medical College, Huazhong University of Science and Technology, Wuhan 430030, China

**Keywords:** acute coronary syndrome, immunosenescence, CD4+ T cells, CD28null T cells, infection

## Abstract

Acquired immune responses mediated by CD4^+^ T cells contribute to the initiation and progression of acute coronary syndrome (ACS). ACS patients show acquired immune system abnormalities that resemble the characteristics of autoimmune dysfunction described in the elderly. This study aimed to investigate the role of premature CD4^+^ T cells senescence in ACS and the underlying mechanism. We compared the immunological status of 25 ACS patients, 15 young healthy individuals (C1), and 20 elderly individuals with absence of ACS (C2). The percentages of CD4^+^ T lymphocyte subsets (including naïve, regulatory, memory and effector T cells) in peripheral blood were analyzed. In ACS patients, a significant expansion of CD4^+^CD28^null^ effector T cells and a decline of CD4^+^CD25^+^CD62L^+^Treg cells were observed. In addition, patients with ACS showed an accelerated loss of CD4^+^CD45RA^+^CD62L^+^ naïve T cells and a compensatory increase in the number of CD4^+^CD45RO^+^ memory T cells. ACS patients demonstrated no significant difference in frequency of T cell receptor excision circles (TRECs) compared to age-matched healthy volunteers. The expression of p16^Ink4a^ was increased while CD62L was decreased in CD4^+^CD28^null^ T cells of ACS patients. Compared to healthy donors, ACS patients demonstrated the lowest telomerase activity in both CD4^+^CD28^+^and CD4^+^CD28^null^ T cells. The serum levels of C-reactive protein, Cytomegalovirus IgG, *Helicobactor pylori* IgG and *Chlamydia pneumonia* IgG were significantly higher in ACS patients. The results suggested that the percentage of CD4^+^ T cell subpopulations correlated with chronic infection, which contributes to immunosenescence. In conclusion, chronic infection induced senescence of premature CD4^+^T cells, which may be responsible for the development of ACS.

The acute coronary syndromes (ACS), including unstable angina and acute myocardial infarction (MI), are mainly caused by the disruption of atherosclerotic plaques and secondary stenosis or occlusion [1]. As the dominant cause of ACS, atherosclerosis is a chronic inflammatory disease of the arterial wall which involves both innate and adaptive immune responses [2]. Inflammation in the atherosclerotic lesions is manifested by the continuous infiltration and activation of immune cells, especially CD4^+^ T cells [3]. In addition to macrophages and cytokines, CD4^+^ T cells have been reported to accumulate in early and advanced human atherosclerotic plaques and contribute to the progression and destabilization of these atherosclerotic lesions [3]. T cells mediate lysis of endothelial cells, and fibrous cap attenuation contribute to the destabilization of atherosclerotic plaques and fatal coronary events [4]. Detailed analysis of CD4^+^ T cells in ACS patients reveals an relatively increased frequency of an unusual subset, CD4^+^CD28^null^ T cells, which are characterized by defective cell surface expression of CD28 [5]. CD4^+^CD28^null^ T cells are considered to be a marker of aging of the immune system [6].

Immunosenescence describes the progressive dysfunction of systemic immunity during aging, which is characterized by more susceptibility to infectious diseases and greater morbidity and mortality[7]. The decline in the output of new cells from the thymus, low numbers of naïve T cells, and compensatory increasing of memory T cells in the peripheral blood of the elderly are generally considered hallmarks of immunosenescence. Infectious agents, such as stimulating antigens, contribute to the persistent activation of T cells and the production of autoimmune antibodies. The stress of persistent immune response and chronic inflammation accelerate immuno-senescence. Premature immunosenescence may lead to disruptions in self-tolerance and play an important role in the pathogenesis of some autoimmune diseases[8].

Patients with ACS also show some abnormalities of the acquired immune system that resemble the typical characteristics of immunosenescence. Research has demonstrated an association between atherosclerosis and chronic infection. For example, human cytomegalovirus (CMV) can cause persistent infections [9], which could potentially cause immunosenescence. In the present study, we hypothesize that ACS patients may have premature immunosenescence, especially CD4^+^ T cell senescence. The frequency of effector T cells, regulatory T cells (TREGs), naïve T cells and memory T cells were examined. The impact of chronic infection on changes of T cell subsets with age was further analyzed. Our results show that dysfunction of immune system is involved in the pathogenesis of ACS.

## MATERIALS AND METHODS

### Study population

Peripheral blood mononuclear cells (PBMCs) were obtained from 25 patients with ACS admitted to the Cardiovascular Department of Tongji Hospital. Thirty-five healthy donors were also evaluated. Healthy donors were divided into a young group (C1, < 30 years old) and an elderly group (C2, >30 years old) (Table 1). All 25 patients fulfilled the criteria for ACS, including angiographically confirmed coronary artery stenosis (≥ 50%) and myocardial ischemia on ECG with increase in serum markers of myocardial infarction (MI). Part of ACS patients were associated with a history of hypertension (18/25) and diabetes (12/25). The C2 group comprised donors with typical chest pain but coronary angiography suggested no significant atherosclerosis (AS) and ECG changes showed no MI, as well as no detection of increase in serum MI markers. None of the donors had a personal or family history of acute or chronic infectious diseases (HIV, HBV, or HCV), malignancy, or immune system disorders. The study was approved by the local ethical committee of the Tongji hospital. All ACS patients and healthy donors gave their written informed consent to participate in this study. Characteristics of the ACS patients and healthy donors are summarized in Table 1.

**Table 1:** Characteristics of the healthy donors and ACS patients.

Parameter	C1 (n = 15)	C2 (n = 20)	ACS (n = 25)	*P* *
Age (years)	26 ± 3	67 ± 5	66 ± 3	0.64
Sex (Female/Male)	7/8	9/11	10/15	0.74
Cholesterol (mmol/L)	-	4.66 ± 1.24	4.23 ± 0.72	0.27
LDL (mmol/L)	-	2.30 ± 1.24	2.66 ± 0.81	0.36
HDL (mmol/L)	-	1.13 ± 0.22	1.08 ± 0.26	0.61
cTnI (ng/mL)	-	0.02 ± 0.01	3.48 ± 5.31	0.024
Ejection Fraction (%)	-	67.86 ± 5.61	56.27 ± 10.99	0.001
History of DM	none	7/13	12/13	0.38
History of HBP	none	15/5	18/7	0.82
Family History of CHD	none	0/20	8/17	0.006
Smoking (ever and current)	none	7/13	8/17	0.83

LDL, low density lipoprotein; HDL, high density lipoprotein; cTnI, cardiac troponin I; DM, diabetes mellitus; HBP, high blood pressure; CHD, coronary heart disease. Values represented as means ± S.D (standard deviation) or ratio. P* means statistical difference between the C2 and ACS groups.

### Isolation of PBMCs

PBMCs were isolated by Ficoll gradient centrifugation using LymphoPrep (Axis Shield, Oslo, Norway) according to the manufacturer’s instructions. Red blood cell (RBC) lysis was performed with RBC Lysis Solution (Guge Bio, China). Subsequently, cells were washed twice with phosphate buffered saline and suspended to a count of 1×10^6^ cells/ml.

### Analysis of T cell subsets

PBMCs (5×10^5^/ml) were incubated for 20 minutes at 4? in the dark with human monoclonal antibodies specific for CD3, CD4, CD45RA, CD25, CD45RO, CD28, and CD62L. Antibodies labeled with fluorescence were obtained from BD Bioscience (San Diego, CA, USA), including phycoerythrin (PE)-conjugated antibodies for CD25 (#555432), CD45RA (#556627), CD45RO (#555493), and CD28 (#555729); Antibodies were obtained from Biolegend (San Diego, CA, USA), including fluorescein isothiocyanate (FITC)-conjugated anti-CD3 (#344804), Percep/Cyanine5.5-conjugated anti-CD4 (#344608), and allophycocyanin (APC)-conjugated anti-CD62L (#304810), respectively. According to manufacturer's instruction, antibodies were diluted as 1:100 per sample and analysis were performed in triplicate. After incubation, cells were washed twice with phosphate buffered saline (PBS) and fixed with 4% paraformaldehyde. All analyses were performed using a BD FACSC flow cytometer with CellQuest software (BD PharMingen). Results were expressed as the percentages of gated lymphocytes. CD3^+^CD4^+^CD28^null^ T cells were characterized as total effector T cells, CD3^+^CD4^+^CD25^+^CD62L^+^T cells were characterized as Treg cells, CD3^+^CD4^+^CD45RA^+^CD62L^+^ T cells were characterized as naïve T cells, and CD3^+^CD4^+^CD45RO^+^ T cells were characterized as memory T cells.

### Fluorescence-activated cell sorting (FACS)

PBMCs were prepared as described above and stained with PE conjugated anti-CD4 and APC conjugated anti-CD28 (Abcam, CA, USA). For sorting on PE (anti-CD4) and APC (anti-CD28), a negative (unstained) control as well single-color controls for each parameter were prepared. Cells were then sorted using a MoFlo cell sorter (DakoCytomation). Based on the relevant expression of CD4 and CD28, CD4^+^CD28^+^ and CD4^+^CD28^null^ T cells were sorted with post-sort purity of 97-99%.

### Construction of plasmid containg TREC gene and DNA standard

DNA was extracted from separated PBMCs using the Universal Genomic DNA Extraction Kit Ver.3.0 (TaKaRa), according to manufacturer’s instructions. All DNA samples were stored at -80?. The TREC gene (392 bp) was cloned into the plasmid vector, pMD 18-T (TaKaRa). The RAG2 gene (476 bp) was subsequently cloned into the EcoR I site of the same plasmid vector. Plasmid purification was conducted by using MiniBEST plasmid Purification Kit Ver 2.0 (TaKaRa). A 10-fold serial dilution was prepared prior to use.

### Real-time PCR assay

Real-time PCR was performed with a PCR Thermal Cycler Dice Real Time System (TaKaRa Code.TP800). The multiplex assay contained the following components in a final volume of 25 ml: 50 ng DNA, 10 mM of each forward and reversed primer, 3 mM probe, and 12.5 ml Premix Ex Taq (2 ×). The cycling program used to show as below: initial denaturation at 95? for 30 seconds, followed by 45 cycles of 95? for 5 seconds, 55? for 10 seconds, and 72? for 20 seconds. Each experiment was performed in duplicate. Signal-joint TREC concentrations were determined by real-time PCR using the TaqMan technique. For quantification, we used the housekeeping gene RAG2 as an internal standard. The ratio of TRECs normalized to RAG2 was used to represent number of TRECs DNA in each sample (1×10^3^ T cells). Moreover, the relative expression level of CD62L and CDK2NA was normalized to GAPDH respectively. Primer and probe sequences are given in Table 2.

**Table 2:** Designs of primers and probes.

Primer	Sequence
RAG2 - forward	5'-GCAACATGGGAAATGGAACTG-3'
RAG2 - reversed	5'-GGTGTCAAATTCATCATCACCATC-3'
RAG2 - probe	5'-(FAM)CCCCTGGATCTTCTGTTGATG TTTGACTGTTT GTGA (Eclipse)-3'
TREC - forward	5'-GGGATGTGGCATCACCTTTG-3'
TREC - reversed	5'-AAGAAGAAGAAGAAGAAGG CTCTG-3'
TREC - probe	5'-(FAM)TTCCCCACAGGAGCC CCATTTT GCC (Eclipse)-3'
SELL - forward	5'-CTGCTGGACTTACCATTAT-3'
SELL - reversed	5'-TATGGCAACTAAATCTGTG-3'
p16^Ink4a^ - forward	5'-CGGAGGCCGATCCAGGTCAT-3'
p16^Ink4a^ - reversed	5'-AGCACCACCAGCGTGTCCAG-3'
GAPDH - forward	5'- AATCCCATCACCATCTTCC-3'
GAPDH - reversed	5'- GGACTCCACGACGTACTCA-3'

The probe was labeled with FAM at the 5'-end and Eclipse at the 3'-end. RAG2, recombination activating gene 2; TREC, T cell receptor excision circles.

### Detection of Telomerase Activity

Telomerase repeated amplification protocol (TRAP) was used to detect telomerase activity. Cell lysis was prepared from FACS-sorted CD4^+^CD28^+^ and CD4^+^CD28^null^ T cells (1×10^4^), respectively. Samples were subsequently analyzed by TRAPeze® Telomerase Detection Kit (Millipore, CA, USA) according to the manufacturer’s instructions. ImageQuant Software was used to determine the intensity of the telomerase products (6 bp-ladder) and the ITAS (Internal Standard Control) band. The ratio of intensity levels of TRAP sample ladders (~50 bp) and ITAS band (36 bp) was calculated. Each sample was normalized to the positive control as a percentage (positive control: samples of the young healthy group) after background subtraction.

### Measurement of C-reactive protein (CRP) activity in serum

Serum samples were used to detect CRP concentrations. CRP measurement, based on competitive enzyme-linked immunosorbent assay (ELISA), was performed by using the Human C-Reactive Protein (CRP) ELISA Kit (eBioscience, USA). According to the manufacturer's instructions, the optical density (OD) was detected at a wavelength of 450 nm using a PHOMO microplate reader.

### IgG antibodies against Helicobactor pylori (HP), Cytomegalovirus (CMV) and Chlamydia pneumonia (Cpn) in serum

Specific ELISA Kits (IBL-International, GmbH, Germany) were used to determine HP, CMV and Cpn-specific IgG in the blood sera of the patients and healthy donors. Samples were run and analyzed with a PHOMO microplate reader using Auto soft.

### Statistical analysis

Statistical analysis was performed with SPSS statistic software package (Version 11.0). Differences of continuous variables between the three groups were examined using one-way analysis of variance (ANOVA). Categorical data were analyzed with a Pearson's chi-squared test (χ^2^test). Correlation analysis was performed using Spearman’s rank correlation coefficient test. P values less than 0.05 are statistically significant.

## RESULTS

### Increased frequency of CD3^+^CD4^+^CD28^null^ effector T cells with advancing age in ACS patients

CD4^+^CD28^null^ T cells represent a minor subset of CD4^+^ T cells in healthy individuals, accounting for about 2.2-6.4% [10]. The expansion of CD4^+^CD28^null^ T cells is associated with aging, infection, and chronic inflammatory diseases [6]. Consistent with the published data[11], our results showed that the CD3^+^CD4^+^CD28^null^T cell subset was rarely observedin healthy young controls, with a frequency of 1.6 ± 1.3% of CD3^+^CD4^+^ T lymphocytes. The frequency increased to 5.4 ± 2.2% in elderly healthy individuals and 12.2 ± 3.8% in ACS patients (p < 0.05, Supplementary Fig. 1).

### Decreased frequency of CD3^+^CD4^+^CD25^+^CD62L^+^ Tregs in ACS patients

Tregs is important for maintaining homeostasis and limiting autoimmune responses. A decline in Treg numbers with age has been well documented [12, 13]. However, we found no significant difference of Treg frequency between young healthy donors and elderly healthy donors (7.4 ± 2.0% vs. 6.4 ± 2.9%, p = 0.26, Fig. 2). The frequency of Treg was significantly decreased in the ACS patients compared to all healthy patients (2.55 ± 0.96%, p<0.05, Supplementary Fig. 2). These results indicate that the dysfunction of Tregs in patients with ACS might be due to decreased amount.

### Decreased frequency of CD3^+^CD4^+^CD45RA^+^CD62L^+^ naïve T cells and compensatory increase of CD3^+^CD4^+^CD45RO^+^ memory T cells in ACS patients

**Figure 1.**
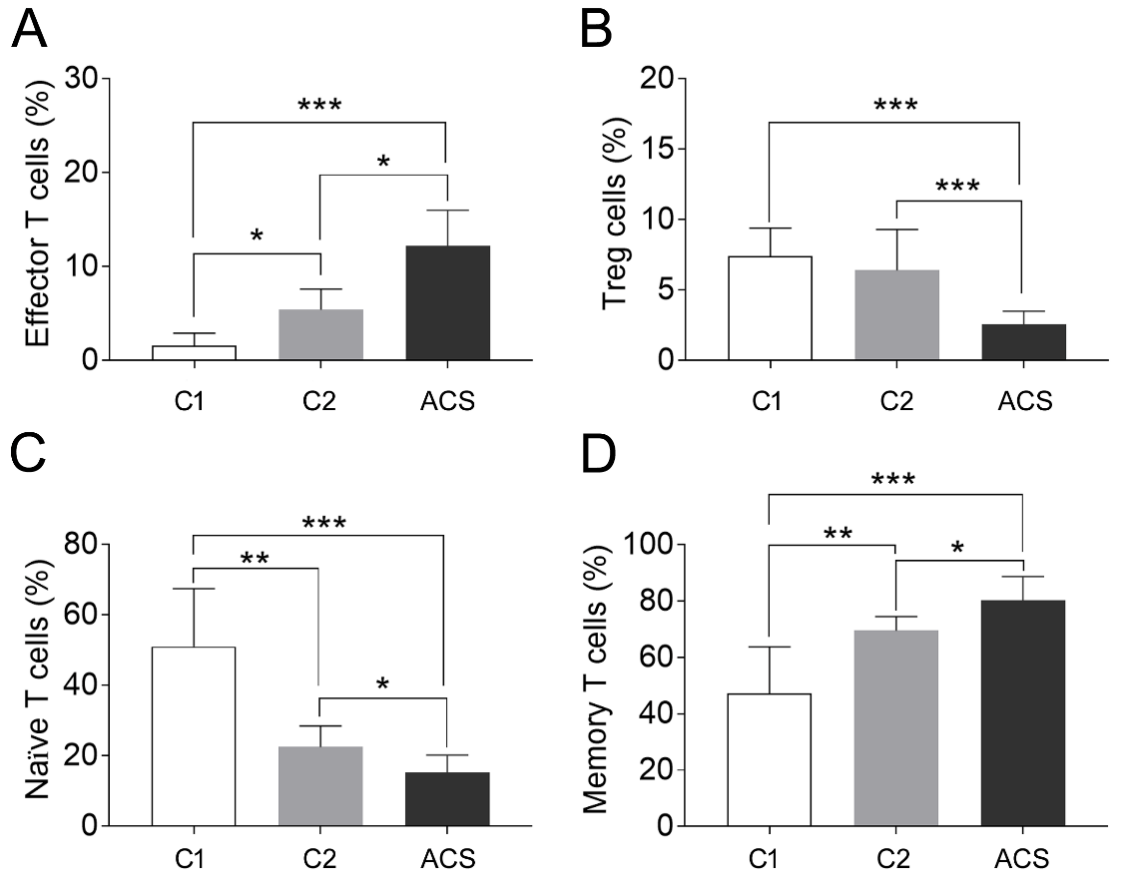
The percentage change of CD4^+^ T cell subpopulations in PBMCs isolated from young healthy donors (C1), elderly healthy donors (C2), and ACS patients. CD3^+^CD4^+^CD28^null^ T lymphocytes was characterized as effector T cells, CD3^+^CD4^+^CD25^+^CD62L^+^T cells was characterized as regulatory T (Treg) cells, CD3^+^CD4^+^CD45RA^+^ T lymphocytes were characterized as naïve T cells, and CD3^+^CD4^+^CD45RO^+^ T cells was characterized as memory T cells. (A) Percentage of CD3^+^CD4^+^CD28^null^ T cells, C1 vs.C2 vs. ACS, 1.6 ± 1.3% vs. 5.4 ± 2.2% vs. 12.2 ± 3.8%. (B) Percentage of CD3^+^CD4^+^CD25^+^CD62L^+^ Treg cells, C1 vs.C2 vs. ACS, 7.4 ± 2.0% vs.6.4 ± 2.9% vs. 2.55 ± 0.96%. (C) Percentage of CD3^+^CD4^+^CD45RA^+^CD62L^+^ T cells, C1 vs.C2 vs. ACS, 51.0 ± 16.5% vs. 22.5 ± 6.0% vs. 15.3 ± 4.9%. (D) Percentage of CD3^+^CD4^+^CD45RO^+^ T cells, C1 vs.C2 vs. ACS, 47.2 ± 16.6% vs. 69.5 ± 5.0% vs. 80.4 ± 8.4%. Values represented as means ± S.E.M (Standard Error of the Mean), n > 10, * *P* < 0.05, ** *P* < 0.01, *** *P* < 0.001.

The two isoforms of CD45, CD45RA and CD45RO, have been suggested to distinguish naïve from memory T cells and characterize the maturity of T cells [14]. Thus, in this study T cell phenotypes were determined from PBMCs as CD3^+^CD4^+^CD45RA^+^CD62L^+^ naïve T cells and CD3^+^CD4^+^CD45RO^+^ memory T cells, respectively. The results showed that the percentage of naïve T cells gradually decreased in PBMCs isolated from the young and elderly healthy donors, and the ACS patients (51.0 ± 16.5% vs. 22.5 ± 6.0% vs. 15.3 ± 4.9%, p < 0.05, Fig. 1). In contrast, the percentage of memory T cells showed an increase in the three corresponding groups (47.2 ± 16.6% vs. 69.5 ± 5.0% vs. 80.4 ± 8.4%, p < 0.05, Fig. 1). These results indicate that memory T cells from ACS patients compensate for the loss of functional naïve T cells, which might contribute to immune disturbance.

### Increased expression of senescence associated gene in CD4^+^CD28^null^ T cells in ACS patients

**Figure 2.**
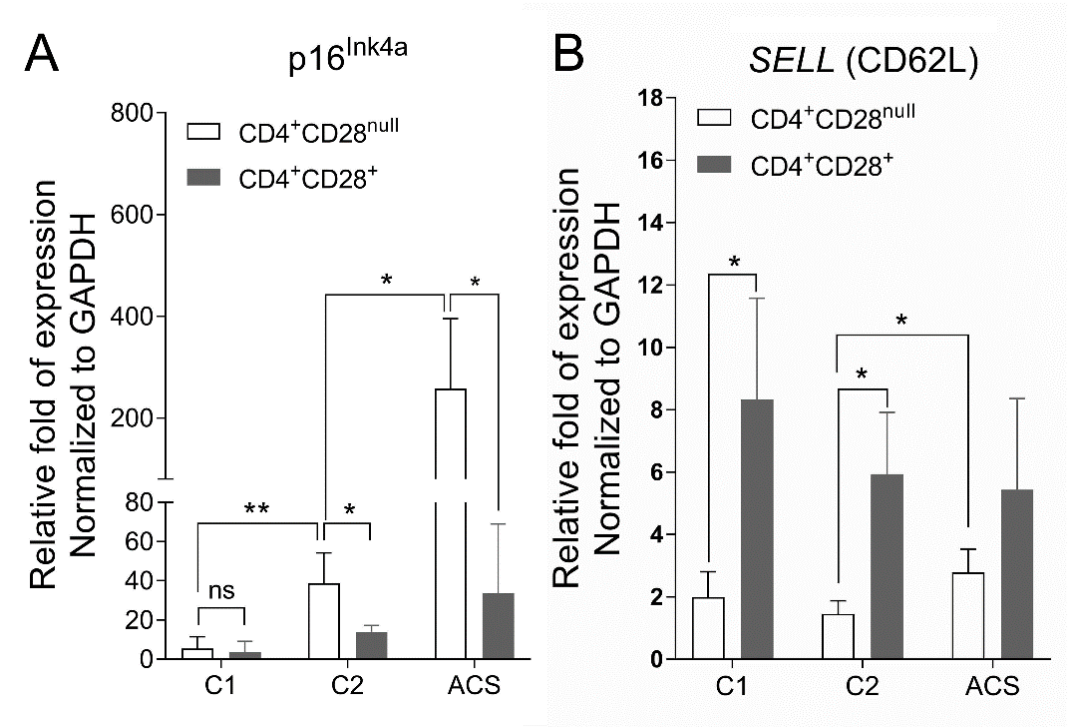
Expression change of senescence associated gene and T cell function marker gene. PBMCs were isolated from peripheral blood of young healthy donors (C1), elderly healthy donors (C2) and ACS patients. CD4^+^CD28^+^ and CD4^+^CD28^null^ T lymphocytes were further sorted and purified by a MoFlo XDP Cell Sorter. Expression levels for p16^Ink4a^ (A) and *SELL* (CD62L) (B) in CD4^+^CD28^+^ and CD4^+^CD28^null^ T cells were measured using quantitative PCR and normalized by GAPDH mRNA, respectively. Values represented as means ± S.D., n > 3, * P < 0.05, ** P < 0.01.

As p16^Ink4a^ apoptosis inhibitor has an established role in cellular senescence of peripheral T cells, we tested whether p16^Ink4a^ expression is associated with expansion of CD4^+^CD28^null^ T cells in elderly healthy donors and ACS patients. Data showed that p16^Ink4a^ expression was increased in two elderly groups compared with that in the young donors (Fig. 2A), which is in line with previous reports on p16^Ink4a^ expression with aging[15]. Moreover, p16^Ink4a^ expression level was significantly higher in CD4^+^CD28^null^than in CD4^+^CD28^+^ T cells when normalized to the expression of housekeeping gene GAPDH. The difference was more remarkable in the ACS patients than in healthy groups. The results suggested that the accumulation of CD4^+^CD28^null^ T cells may partially explained by their increased resistance to apoptosis.

### Decreased expression of circulating marker on CD4^+^CD28^null^ T cells in ACS patients

CD62L (L-selectin) is a surface marker on circulating naïve T cells during homeostasis. To investigate whether peripheral CD4^+^ T lymphocytes from ACS patients have the same recirculation efficiency and property, we investigated expression of *SELL*, the gene coding for CD62L, on *ex vivo* isolated CD4^+^ T cells from human peripheral blood. After normalization by GAPDH, expression levels of *SELL* on CD4^+^ T cells were compared among young healthy group, aging healthy group and aging ACS patients. The difference in *SELL* transcript expression level was very dramatic between CD28^+^ and CD28^null^ fractions of CD4^+^ T cells from different donor groups (Fig. 2B). Specifically, young CD4^+^ T-cell populations were dominated by CD28^+^cells, presumably naïve T cells, which expressed high level of *SELL* gene. As donors aged, *SELL* expression on CD4^+^CD28^+^ T cells reduced, and it was comparable on the same lymphocyte subpopluation from age-matched ACS patients (Fig. 2B). In contrast, the expression of *SELL* by specifically sorted CD4^+^CD28^null^ T cells, possibly non-lymph node homing effector T cells, slightly increased in ACS patients (Fig. 2B). These results suggest that age-related exhaustion of naïve CD4^+^ T cells could be related to a reduced *SELL* (CD62L) expression among CD28^+^ cells. Up-regulation of *SELL* might be responsible for recirculation of CD4^+^CD28^null^ T-cells due to alteration of the inflammatory condition with ACS.

### Decreased telomerase activity in CD4^+^ T cells in ACS patients

**Figure 3.**
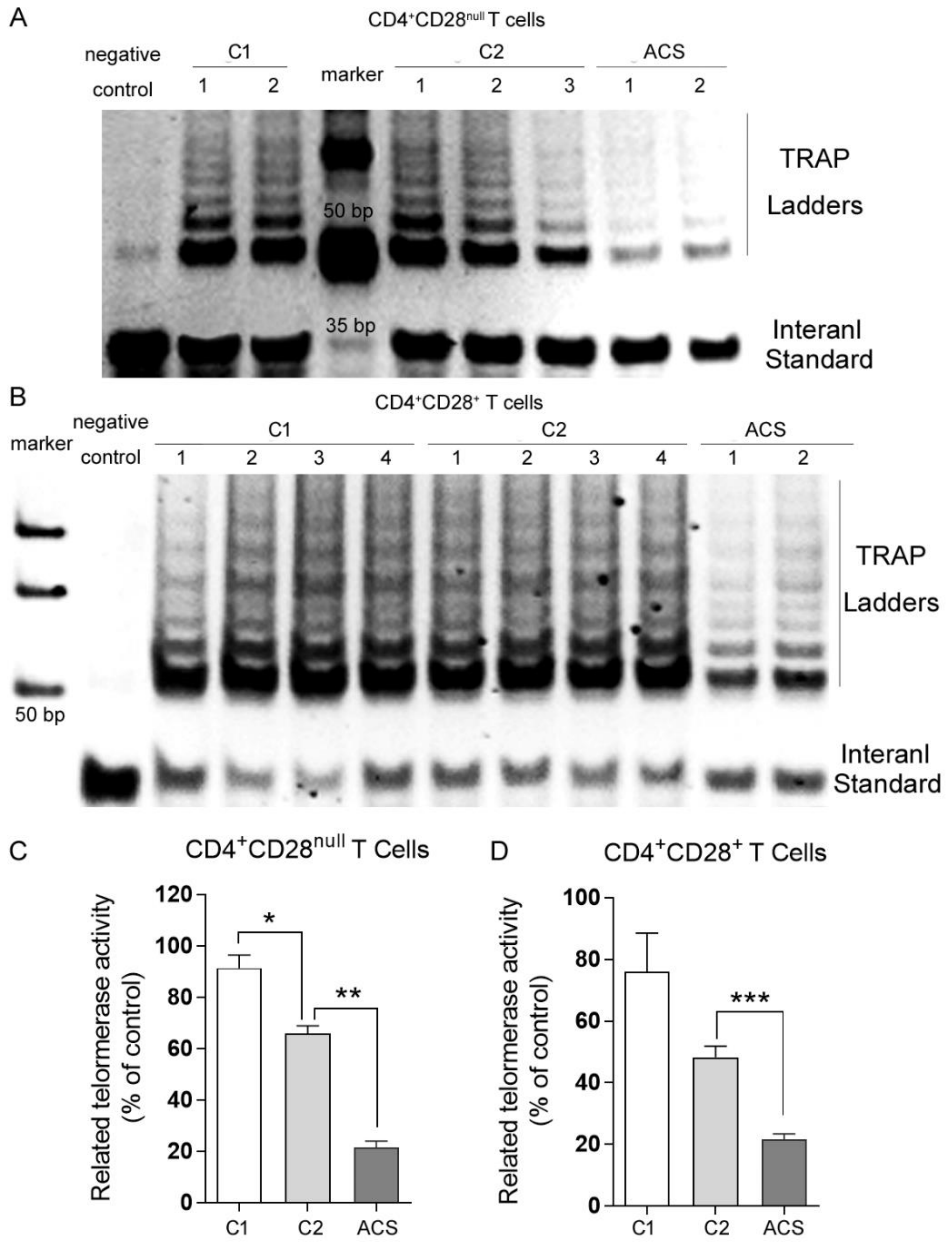
Telomerase activity analysis. CD4^+^CD28^+^ and CD4^+^CD28^null^ T lymphocytes were sorted and collected as described. Telomerase repeated amplification protocol (TRAP) was used to detect telomerase activity. CD4^+^CD28^null^ (A) and CD4^+^CD28^+^ (B) T lymphocytes from indicated sample groups were loaded in lanes as showed, respectively. No cells were added in lysis buffer as a negative control. (C) and (D) ImageQuant Software was used to determine the intensity of the telomerase products and the ITAS (Internal Standard Control) band. The intensity ratio of levels of TRAP sample ladders (~50 bp) and ITAS band (36 bp) was calculated. Each sample was normalized to a young healthy donor as a percentage after background subtraction. Values represented as means ± S.D., n > 3, * P < 0.05, ** P < 0.01, *** P < 0.005.

High level of cellular telomerase activity generally reflects the capacity of overcoming telomere shortening and maintaining limitless cell division. To distinguish ACS pathology from natural age-related decline, telomerase repeated amplification protocol (TRAP) was used to detect telomerase activity in CD4^+^ T cells sorted from healthy donors and ACS patients. Telomerase activity (TA) was measured as intensity ratio of TRAP ladder (~ 50 bp) to ITAS band (36 bp) and normalized as described in *materials and methods*. From the same donors, CD4^+^ T cells were further divided according to CD28 expression: CD28^+^(presumably naïve) versus CD28^null^ (presumably late differentiated) cells. The naïve cells showed higher and CD28^null^ cells showed lower TA (Fig. 3A and 3B). This is in accordance with the established CD4^+^ T cell differentiation pathway and confirms that CD28^null^ T cells as effector cells approaching senescence. As normal aging, healthy donors showed significantly lower TA in CD28^null^ cells (p < 0.05, Fig. 3A and 3C), while they showed moderately lower TA in CD28^+^cells. ACS patients demonstrated the lowest TA in both CD28^+^and CD28^null^ T cells (*p* < 0.01, Fig. 3). In fact, the extent of TA reduction was more significant in ACS patients than in healthy donors with normal aging (*p* < 0.01, Fig. 3). These data indicated that except for cellular differentiation and donor aging, infections and/or inflammation have profound but independent effects on telomerase activity of CD4^+^ T lymphocyte subpopulations, implying distinct mechanisms and consequences on the immune system. Immunosenescence is strongly driven by persistent infections and tissue inflammation.

### No significant reduction of TREC numbers in T lymphocytes in ACS patients

TRECs are stable DNA episomes formed during T cell receptor rearrangement. They are not replicated. They decrease in number through the process of cell divisions, and thus can be used as an indicator of recent thymic emigrants. The numbers of TRECs per 10^3^ T cells suggested that no obvious change of TRECs concentration in total peripheral T lymphocytes occurred with aging (1.20 ± 0.35 vs. 1.20 ± 0.27, Table 3), while a slight decline could be observed in the elderly ACS individuals (1.20 ± 0.27 vs. 1.08 ± 0.16, p = 0.26, Table 3).

**Figure 4.**
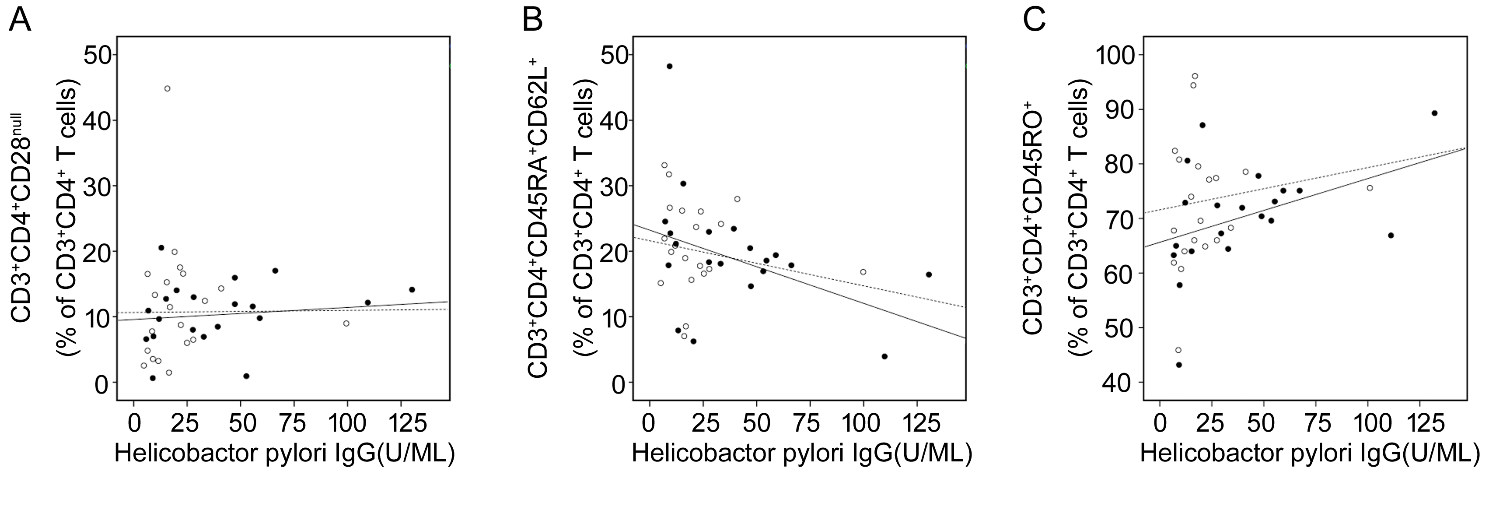
Serum H. pylori LgG concentrations were correlated with T cell subsets isolated from PBMSc. The correlations of CD3^+^CD4^+^CD28^null^ (A), CD3^+^CD4^+^CD45RA^+^CD62L^+^ (B) and CD3^+^CD4^+^CD45RO^+^ (C) T cells with serum HP IgG concentrations in healthy elderly donors (C2) and ACS patients were shown respectively. Open circles and dashed line represent C2; solid circles and solid line represent ACS patients. (A) For C2, r = 0.132, P = 0.349; for ACS patients, r = 0.593, P = 0.016. (B) For healthy controls, r = -0.492, P = 0.062; for ACS patients, r = -0.664, P = 0.009. (C) For C2, r = 0.629, P = 0.019; for ACS patients, r = 0.709, P = 0.011.

### Elevated levels of chronic inflammatory markers in patients with ACS

Epidemiological studies have suggested that host immune reaction against persistent infectious pathogens such as CMV, HP, and Cpn may promote the development of atherosclerosis [16]. We found that CRP levels were significantly higher in serum from the groups of aged healthy donors and ACS patients compared to those from the group of young healthy donors (Table 3). Accordingly, higher levels of serum IgG antibodies against CMV, HP, and Cpn were detected in patients with ACS (Table 3).

**Table 3:** Numbers of TRECs in T lymphocytes and chronic inflammatory levels in young healthy (C1), elderly healthy (C2) and ACS individuals.

	C1 (n = 15)	C2 (n = 20)	ACS (n = 25)	*P* *
TRECs (per 10^3^ T cells)	1.20 ± 0.35	1.20 ± 0.27	1.08 ± 0.16	0.87
CRP (mg/L)	0.20 ± 0.16	1.36 ± 1.15	2.40 ± 0.86	0.001
CMV IgG (+/-)	13/2	20/0	25/0	0.65
HP IgG (U/ML)	3.95 ± 1.16	11.88 ± 6.47	37.98 ± 32.19	0.001
Cpn IgG(+/-)	4/11	14/6	22/3	0.001

ACS, acute coronary syndrome; CRP, C-reactive protein; CMV, cytomegalovirus; HP, Helicobactor pylori; Cpn, Chlamydia pneumonia. One-way ANOVA was used to compare continuous variables among the three groups. P means the statistical differences between the combined the C1, C2 and ACS groups.

### Correlations of CD3^+^CD4^+^CD28^null^, CD3^+^CD4^+^CD45RA^+^CD62L^+^ and CD3^+^CD4^+^CD45RO^+^ T cells with serum anti-HP IgG antibody concentration in elderly healthy donors and ACS patients

In ACS patients, as shown above, serum levels of IgG against specific microbial antigens increased, accompanied by significant changes in the composition of the CD4^+^ T-cell repertoire. Further analysis showed that the expansion of CD3^+^CD4^+^CD28^null^ T cells (Fig. 4A) and the compensatory increase of CD3^+^CD4^+^CD45RO^+^ T cells (Fig. 4C) were significantly associated with the concentrations of HP-IgG in the blood samples from both healthy donors and ACS patients. Meanwhile, the decline of CD3^+^CD4^+^CD45A^+^CD62L^+^ T cells were correlated with the serum levels of HP-IgG (Fig. 4B). Moreover, the frequencies of T cells subsets were more closely correlated with serum HP-IgG levels in the group of ACS patients than in the group of healthy donors. The results suggested that chronic infection and inflammation is involved in the occurrence of immunosenescence within ACS elderly individuals.

## DISCUSSION

It has been widely accepted that innate and acquired immunity are involved in the initiation and progression of atherosclerotic lesions. Together with a chronic inflammatory response, stable plaque in AS patients may develop into a vulnerable plaque, which triggers some acute diseases such as ACS. However, it is currently unclear how immunologic changes result in the progression of AS and chronic inflammation within atherosclerotic plaques.

This study demonstrated a decrease of naïve T cells with compensatory increase of memory T cells, as well as an expansion of CD4^+^CD28^null^ T cells with the decline of Tregs. These changes suggest a premature senescence of CD4^+^ T cell subsets in ACS patients, compared to age-matched healthy individuals. Moreover, the higher circulating level of CRP and pathogenic IgG indicated chronic inflammation and infection was involved in the process of ACS.

CD4^+^ T cells, as the major component of adaptive immunity, protect humans from infection, and have a declining ability to respond with increasing age [17]. Senescence of CD4^+^ T cells makes ACS patients more susceptible to pathogenic infection, which can explain why chronic infection always accompanies ACS [18]. Infectious pathogens act as a stimulator for activation and differentiation of T cells from naïve to end stage. Together with the decreased regenerative capacity of the thymus, exhaustion of naïve cells and accumulation of effector T cells in ACS patients result in an aging-characterized immuno-phenotype. End-differentiated T cells, such as CD4^+^CD28^null^ T cells are mainly pro-atherosclerotic [19]. They could avoid the diminished suppressive effect of Tregs and promote inflammation in plaques by secreting cytokines. Thus, the imbalance between pro-atherogenic and regulatory lymphocytes and their inflammatory reaction results in plaque progression and destabilization [20]. However, a small portion of healthy individuals have high numbers of CD4^+^CD28^null^ T lymphocytes [21]. This can occur because in healthy individuals, regulatory T cells keep their crucial function of maintaining immune homeostasis, and inflammatory CD4^+^CD28^null^ T cells can be suppressed by Tregs. Clinical trials have shown no significant benefit of antibiotics to AS patients. This suggests that infection is an early comorbidity of AS, triggering disturbance of T cells and initial inflammatory effects. Once the inflammation occurs, it becomes independent of the initiation process and progresses into plaque instability. The increased presence of potentially pathogenic CD28^null^CD4^+^ T cells might be a clinical marker for ACS diagnosis and a promising target for immunotherapy.

The frequency of TRECs, a conserved marker of thymus output, has been found to be age-inappropriately diminished in many immune-associated diseases, such as RA and multiple sclerosis [22]. In this study, we found no significant reduction of TRECs in ACS patients compared with age-matched controls. This may be because the frequency of TREC-positive cells declines between ages 15 and 20 years, corresponding to the time of thymic involution [22], but ACS patients and elderly donors were 60 to 75 years old. Thus, the difference of TREC numbers was less obvious in the elderly. TREC numbers are influenced by multiple factors, including cell division, longevity of the naïve T cells, and intracellular degradation [23]. Our results indicate that accelerated peripheral T cell division rather than decreased thymic output induced the slight decline in TREC levels within T cells in ACS patients.

Our results could explain why ACS most commonly occurs in the elderly. As age increases, immune-senescence increases host susceptibility to microbial pathogens, such as CMV, HP and Cpn. Via a mechanism called molecular mimicry, endogenous HSP60 expressed on the surface of endothelial cells may be recognized by the immune cells stimulated by infectious pathogens [24, 25]. Autoimmune response may be induced because of the high degree of homology between microbial and human antigens. Thus, persistent exposure to infectious agents or pathogens can trigger the activation and differentiation of T lymphocytes toward pro-inflammatory subsets. Imbalance among the T-cell subsets promotes a chronic inflammation and results in destabilization of the plaques.

### Conclusion

From the perspective of immunosenescence, here we described the changes of CD4^+^ T lymphocyte subpopulations and their correlation with infection and chronic inflammation. Composition of CD4^+^ T-cell subsets might mainly be affected by stress from pathogen stimulation in patients with ACS. Our results indicated that central immunosenescence was not dominant in ACS patients. Peripheral immunosenescence caused by chronic infection may lead to immune dysfunction and excessive activation of T cells. A number of research and our previous studies showed that T lymphocyte disorders can produce large amounts of inflammatory cytokines, which cause atherosclerotic plaque rupture and play a crucial role in driving ACS occurrence and progression. This study showed that immunosenescence of T lymphocytes, especially CD4^+^CD28^null^ subpopulation, was involved in the pathogenesis of ACS, which might suggest possibility to develop novel therapies.

## Supplementary Materials

The Supplemenantry data can be found online at: www.aginganddisease.org/EN/10.14336/AD.2020.0203.
