# Supplementary material for: Premature CD4+ T Cells Senescence Induced by Chronic Infection in Patients with Acute Coronary Syndrome
Source: Aging Dis. 2020 Dec 1;11(6):1471–80. doi: 10.14336/AD.2020.0203 (PMC7673853; doi:10.14336/AD.2020.0203)
Supplement: Supplementary file 1 — The Supplemenantry data can be found online at: www.aginganddisease.org/EN/10.14336/AD.2020.0203. [file AD-11-6-1471-s.pdf]

SUPPLEMENTARY DATA

Premature CD4$^+$ T Cells Senescence Induced by Chronic Infection in Patients with Acute Coronary Syndrome

Ming Cao, Lei Ruan, Yi Huang, Jinli Wang, Jinhua Yan, Yu Sang, Shanshan Li, Guan Wang, Xiaofen Wu*

© 2020. Cao M et al. Published online at http://www.aginganddisease.org/EN/10.14336/AD.2020.0203

SUPPLEMENTARY DATA

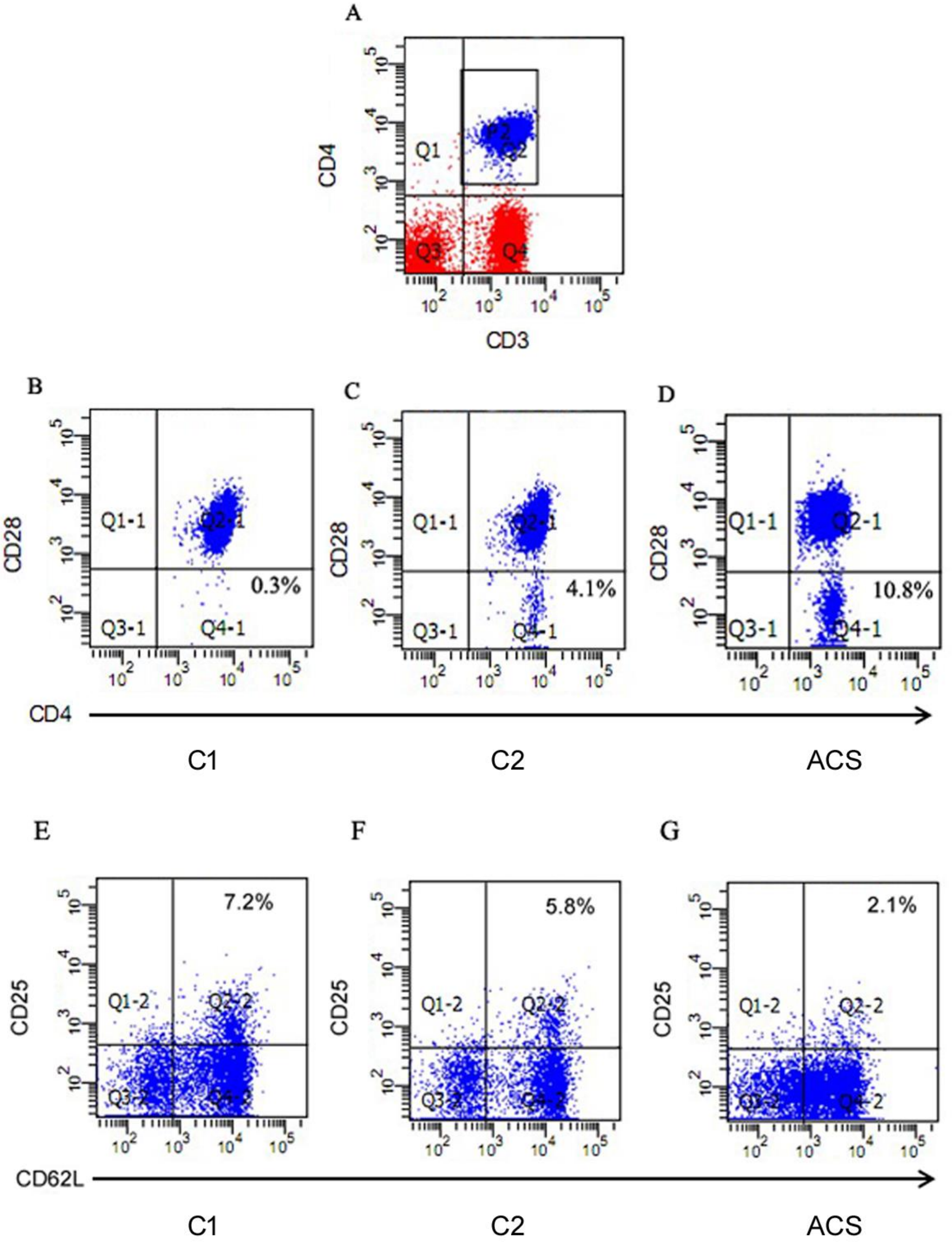

Supplementary Figure 1. Increased percentage of CD3+CD4+CD28null effector T cells and decreased percentage of CD3+CD4+CD25+CD62L+ Treg cells with advancing age in ACS patients. PBMCs were incubated with different antibodies to identify T cell subsets. **(A)** CD3+CD4+ T cells were gated for further analysis. **(B, C and D)** The frequencies of CD3+CD4+CD28null effector T cells were examined from gated cells isolated from a young healthy donor (B, 0.3%), an elderly healthy donor (C, 4.1%), and an ACS patient (D, 10.8%), respectively. **(E, F and G)** The frequencies of CD3+CD4+CD25+CD62L+ Treg cells were determined from gated cells that were isolated from a young healthy donor (E, 7.2%), an elderly healthy donor (F, 5.8%), and an ACS patient (G, 2.1%), respectively.

© 2020. Cao M et al. Published online at http://www.aginganddisease.org/EN/10.14336/AD.2020.0203

SUPPLEMENTARY DATA

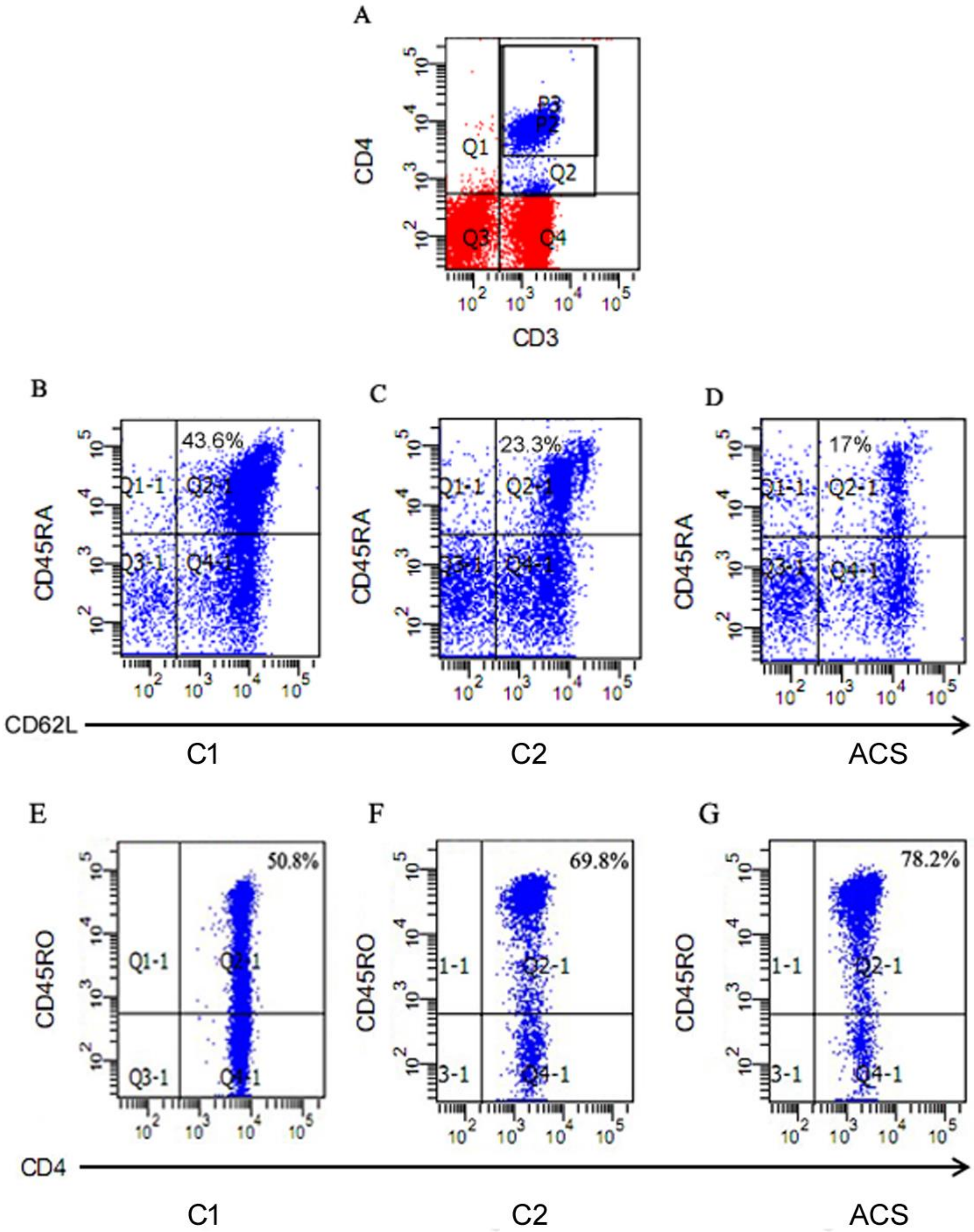

Supplementary Figure 2. Decreased percentage of CD3+CD4+CD45RA+CD62L+ naïve T cells and compensatory increase of CD3+CD4+CD45RO+ memory T cells with advancing age in ACS patients. **(A)** CD3+CD4+ T cells were gated and selected for further analysis. **(B, C and D)** The frequencies of CD3+CD4+CD45RA+CD62L+ naïve T cells were determined from gated T cells that were isolated from a young healthy donor (B, 43.6%), an elderly healthy donor (C, 23.3%), and an ACS patient (D, 17%) , respectively. **(E, F and G)** The frequencies of of CD3+CD4 +CD45RO+ memory T cells were determined from gated populations that were isolated from a young healthy donor (E, 50.8%), an elderly healthy donor (F, 69.8%), and an ACS patient (G, 78.2%), respectively.

© 2020. Cao M et al. Published online at http://www.aginganddisease.org/EN/10.14336/AD.2020.0203
